# Body mass index and cholesterol level predict surgical outcome in patients with hepatocellular carcinoma in Taiwan - a cohort study

**DOI:** 10.18632/oncotarget.8312

**Published:** 2016-03-24

**Authors:** Ya-Ling Lee, Wan-Chun Li, Tung-Hu Tsai, Hsin-Yu Chiang, Chin-Tsung Ting

**Affiliations:** ^1^ Institute of Public Health and Community Medicine Research Center, National Yang-Ming University, Taipei, Taiwan; ^2^ Department of Dentistry, Taipei City Hospital, Taipei, Taiwan; ^3^ Department of Dentistry, School of Dentistry, National Yang-Ming University, Taipei, Taiwan; ^4^ Institute of Oral Biology, School of Dentistry, National Yang-Ming University, Taipei, Taiwan; ^5^ Department of Education and Research, Taipei City Hospital, Taipei, Taiwan; ^6^ Institute of Traditional Medicine, School of Medicine, National Yang-Ming University, Taipei, Taiwan; ^7^ Division of Gastrointestinal Surgery, Department of Surgery, Ren-Ai Branch, Taipei City Hospital, Taipei, Taiwan

**Keywords:** hepatocellular carcinoma, body mass index, serum cholesterol level, curative surgical resection, prognostic indicator

## Abstract

Curative surgical resection (CSR) remains the most effective therapeutic intervention for patients with hepatocellular carcinoma (HCC); however, frequent post-surgical recurrence leads to high cancer related mortality. This study aimed to clarify the role of body mass index (BMI) and serum cholesterol level in predicting post-surgical outcomes in HCC patients after CSR. A total of 484 HCC patients including 213 BMI^high^ and 271 BMI^low^ patients were included. Overall survival (OS) and recurrence-free survival (RFS) rates were examined in patients with differential BMI and serum cholesterol level. The analysis showed that significant different 1-, 3- and 5-year cumulative OS rates (*P-value*=0.015) and RFS rate (*P-value*=0.010) between BMI^low^ and BMI^high^ patients. Further analysis in groups with differential serum cholesterol levels among BMI^low^ and BMI^high^ patients indicated that the BMI^low^/Chol^low^ patients exhibited the significant lower cumulative OS and RFS rates in comparison with the remaining subjects (*P-value*=0.007 and 0.039 for OS and RFS rates, respectively). In conclusion, the coexistence of low BMI and low serum cholesterol level could serve as prognostic factors to predict post-operative outcomes in HCC patients undergoing surgical hepatectomy.

## INTRODUCTION

Hepatocellular carcinoma (HCC) is the fifth most common cancer and the third leading cause of cancer-related deaths worldwide [1]. Hepatic resection remains one of the most common, effective, and widely accepted therapeutic interventions for patients with HCC [2, 3]. However, frequent recurrence of HCC in patients undergoing surgical treatment procedure, such as curative hepatic resection, still results in high cancer related mortality. A recent study has shown that a number of risk factors, such as preoperative serological C-reactive protein (CRP) level, correlated with cancer recurrence in patients with HCC after hepatic resection, revealing the possibility to predict surgical outcome by preoperative clinical physiological parameters [4]. To simplify clinical procedure, the candidate index to predict surgical outcome would need to be common and easy to interpret. While body mass index (BMI) is the most easily accessible information from routine checkouts, testing the correlation of patients' BMI and HCC prognosis is of great interest.

Although obesity has been recognized as a potential life-threatening epidemical risk factor globally, previous epidemiological evidence for the association of BMI and primary HCC incidence and correlation of obesity and HCC prognosis remains inconclusive [5, 6]. It was shown that obesity is associated with an increased incidence of multiple types of cancers as well as an increased perioperative morbidity and mortality in patients undergoing major surgical procedures [7]. Several studies have shown that greater HCC prevalence and patient mortality are associated with a high BMI whereas poorer perioperative outcomes with hepatectomy was observed in HCC subjects with greater BMI [8-12]. In contrast, other groups demonstrated that overweight (BMI ≥ 25) was not a risk factor for surgical outcomes after hepatic resection in patients with primary HCC [13]. The reasons behind these conflicting data might possibly be due to the discordant classification of obesity status among different studies and lack of large sample sized prospective cohort-based investigations. In addition, the fact that other confounding molecular cues, including elevated levels of insulin-like growth factors [14], leptin [15], hormones [16] and cytokines [17] were not adjusted in studies, could also result in the divergent interpretations for prognosis after hepatectomy between lean and obese subjects. With a causal link between BMI and total cholesterol level in many cancers, it was interestingly found an inverse correlation between preoperative total cholesterol levels and clinical postoperative outcomes of HCC patients in several studies [18-20]. Further examination in these studies suggested that poorer surgical outcome in patients with lower preoperative total cholesterol level and BMI might be caused by long-term liver dysfunction and malnutrition implying the potential role of lipid composition for determination of operative outcomes in HCC patients.

Taken together, we hypothesized herein that preoperative BMI and serological cholesterol level could possibly predict long-term outcome in HCC patients undergoing curative treatment. To test this hypothesis, a prospective cohort-based database of patients with primary HCC undergoing hepatic resection surgery from 2000 to 2010 was retrospectively examined. While BMI is considered as a good index for the prediction of medically significant obesity according to National Institutes of Health Consensus Development Conference (NIHCDC) [21], the obesity associated confounding factors including lipid profile and tumor stage were adjusted to better define the correlation between BMI / total cholesterol level and postoperative outcomes in HCC patients.

## RESULTS

### Differential preoperative clinical information of HCC patients with different BMI

Using 25 as a cutoff value, 271 HCC patients (56%) were classified as BMI^low^ and 213 HCC patients (44%) were in the BMI^high^ group. The demographic data and preoperative laboratory examination of these two groups are summarized in Table 1. Among all readouts, there is no significant difference between two groups for age, gender, blood glucose and the preoperative liver function parameters including serum AFP, serum albumin, total bilirubin concentration, AST activity. The tumor characteristics such as grades of tumor size, number, Child-Pugh stage, TNM classification, differentiation status, vascular invasion and UICC stage also did not significantly differ between BMI^low^ and BMI^high^ groups. It is also noteworthy that the total serum cholesterol level was significantly lower in BMI^low^ group compared with BMI^high^ group (*P*-value = 0.029).

**Table 1:** Demographic and laboratory data of participants prior to hepatic resection surgery

Characteristics	BMI^low^ (<25)	BMI^high^ (≥ 25)	Total (N)	*P*-value
**Age (years)**	61.5	61.8	61.6 (484)	0.049^*^
**< 57**	88	68	156	
**≥ 57**	183	145	328	0.896
**Gender**			484	
**Male**	202	161	363	
**Female**	69	52	121	0.792
**DM status**			484	
**Non-DM**	209	142	351	
**DM**	62	71	133	0.011^*^
**Hepatitis**			484	
**B**	193	158	351	
**C**	78	55	133	0.469
**Serum AFP (μg/L)**	4259.7	4997.2	4589.8 (467)	0.818
**≤ 400**	202	167	369	
**> 400**	56	42	98	0.671
**Total bilirubin (mg/dl)**	1	1.23	1.1 (479)	0.263
**< 1.2**	199	153	352	
**≥ 1.2**	69	58	127	0.668
**Serum albumin (mg/dl)**	3.87	3.95	3.9 (475)	0.062
**<3.5**	46	31	77	
**≥ 3.5**	129	179	398	0.446
**Blood glucose (mg/dl)**	107.7	119.8	113.1 (476)	0.142
**≥ 126**	47	42	89	0.489
**AST (IU/mL)**	58.9	51.4	55.6 (481)	0.084
**< 40**	127	108	235	
**≥ 40**	242	104	246	0.416
**Total cholesterol (mg/dl)**	163.9	168.9	166.1 (394)	0.149
**< 200**	191	135	326	
**≥ 200**	30	38	68	0.029^*^
**Child stage**			437	
**A**	206	172	378	
**B**	37	20	57	
**C**	2	0	2	0.153
**TNM stage**			437	
**I**	66	57	123	
**II**	104	88	192	
**III**	45	40	85	
**IV**	27	10	37	0.165
**Differentiation**			430	
**Well**	17	11	35	
**Moderate**	136	93	246	
**Poor**	86	87	199	0.131
**Size (cm)**			484	
**< 5**	182	154	336	
**≥ 5**	89	59	148	0.223
**Number**			484	
**Single**	228	180	408	
**Multiple**	43	33	76	0.911
**Vascular invasion**			379	
**Positive**	143	110	253	
**Negative**	67	59	126	0.537

The results indicated that the obese patients have a high diabetic incidence and high cholesterol levels as others factor showed no significant difference between BMI^high^ and BMI^low^ groups.

* P-value < 0.05.

### BMI is significantly correlated with surgical outcome after curative hepatectomy

**Figure 1:**
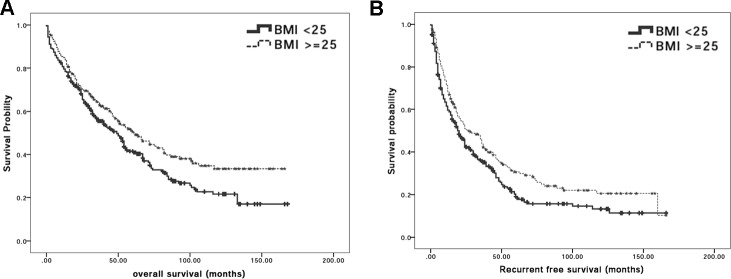
BMI is significantly correlated with surgical outcome after curative hepatectomy **A.** Kaplan-Meier survival curves for overall survival in HCC patients who received hepatectomy according to BMI classification. The cumulative overall survival rates were significantly lower in the BMI^low^ group patients than BMI^high^ group patients (log-rank test; *p* = 0.015); **B.** Kaplan-Meier survival curves for recurrence-free survival in HCC patients who received hepatectomy according to BMI classification. The cumulative recurrence-free survival rates were significantly lower in BMI^low^ than BMI^high^ group (log-rank test; *P*-value = 0.010).

To clarify the association between BMI and surgical outcome, OS and RFS rates in BMI^low^ and BMI^high^ groups were analyzed. Hepatic resection procedure was performed and differential OS rates after surgical treatment between BMI^low^ and BMI^high^ groups were firstly examined. Among all 484 patients, 291 (60.1%) patients including 173 BMI^low^ patients and 118 BMI^high^ patients died during follow-up period. The Kaplan-Meier OS curve of patients between the two groups is plotted (Figure 1A). The respective OS rates at the 1^st^, 2^nd^, 3^rd^, 4^th^ and 5^th^ year were 79.6%, 68.2%, 55.5%, 50.2%, and 41.1% for BMI^low^ group while 84.9%, 70.5%, 63.9%, 56.6%, and 50.5% for BMI^high^ patients. Further analysis for cumulative survival rates indicated that OS rates were significantly lower in the BMI^low^ than in the BMI^high^ population (*P*-value = 0.015).

HCC recurred in 351 patients comprising of 205 BMI^low^ and 146 BMI^high^ patients. Kaplan-Meier survival curves for RFS in all patients are shown in Figure 1B. While the RFS rates at the 1^st^, 2^nd^, 3^rd^, 4^th^ and 5^th^ year were 59.6%, 43.1%, 35.7%, 26.3%, and 19% for BMI^low^ patients and 68.4%, 51%, 43.9%, 35.4%, and 30.2% for BMI^high^ patients, respectively, it was found that the cumulative RFS rate was significantly lower in BMI^low^ than in BMI^high^ group (log-rank test; *P*-value = 0.010) suggesting that lower preoperative BMI might have advert influence for surgical prognosis after liver resection surgery.

### Serum cholesterol level is a potential risk factor for BMI associated surgical outcome after hepatectomy

Potential risk factors that contributed to post-operative cancer recurrence rate after surgical resection were then examined. A univariate analysis identified a number of factors correlated with differential HCC prognosis after surgical treatment. The preferential preoperative parameters for poorer survival rate in patients undergoing liver surgery included: (1) age over 57; (2) BMI < 25; (3) gender (male); (4) abnormal serum AST level; (5) hypoalbuminemia (serum albumin level < 3.5mg/dL); (6) hyperbilirubinemia (serum bilirubin > 1.2mg/dL) (7) higher serum AFP (AFP > 400mg/dl); (8) higher Child-Pugh grading (with a Child-Pugh grade of B or C); (9) poorly differentiation; (10) higher TNM stage grading; (11) greater tumor size ( > 5 cm); (12) the detection of vascular invasion and (13) multiple tumors. In contrast, by using multivariate analysis, it was found that only BMI < 25, hypoalbuminemia, higher serum AFP level, greater TNM stage grading and poorer differentiation were potential risk contributors for HCC prognosis after surgical treatment (Table 2A). Risk factors associated with OS rates after hepatic surgical resection were also examined. A univariate analysis defined 11 identical factors to influence HCC recurrence (Table 2B) whereas the multivariate analysis revealed that BMI < 25, hypoalbuminemia, higher serum AFP level, higher TNM stage grading and higher Child-Pugh grading (with a Child-Pugh grade of B or C) were significant factors contributing to patient survival after surgical procedure (Table 2B).

**Table 2:** Exploration of potential risk factors for post-operative (A) HCC recurrence and (B) OS rate according to univariate and multivariate proportional hazard models

A					B				
Variable	Univariate Cox-regression		Multivariate Cox-regression		Variable	Univariate Cox-regression		Multivariate Cox-regression	
	HR(95% CI)	*P* -value	HR(95% CI)	*P*-value		HR(95% CI)	*P* ·value	HR(95% CI)	*P* -value
**Age (57yrs)**	1.26(1.01~1. 58)	0.046*	1.2(0.91~1.58)	0. 19	**Age (57yrs)**	1.29(1.01~1.66)	0.046*	1.25(0 .93~1 66)	0.133
**Gender (Female)**	0.78(0.04~0.98)	0.036*	1.9(0.68~1 2)	0 .476	**Gender (Female)**	0.76(0.61~1.01)	0.058	-	-
**Obesity (BMI>25Kg/m^2^)**	0.76(0.61~1.94)	0.011^*^	0.76(0. 59~0.98)	0.032^*^	**Obesity (BMI>25Kg/m^2^)**	0.75(0.59~0.95)	0.016*	0.73(0.55~0.97)	0.031^*^
**Diabetes (Y/N)**	0.82(0.81~1.3)	0.815	-	-	**Diabetes (Y/N)**	0.93(0.72~1.21)	0.596	-	-
**Etiology (HCV/others)**	1. 23(0.98~1. 55)	0. 072	-	-	**Etiology (HCV/others)**	1.15(0.9~1.47)	0.273	-	-
**Serum AST value(>401U/L)**	1.52(1.23~1.88)	<0.001^*^	1. 27(0.98~1.65)	0.067	**Serum AST value(>40IU/L)**	1.61(1.27-2.03)	<0.001^*^	1. 26(0.95~1.68)	0.11
**Sugar(>126mg/dl)**	1. 26(0.97~1.64)	0.088			**Sugar(>126mg/dl)**	1.06(0.78~1.43)	0.714	-	-
**Serum albumin(>3.5mg/dl)**	0.49(0.37~0.64)	<0.001^*^	0.46(0.31~0..68)	<0.001^*^	**Serum albumin(>3.5mg/dl)**	0.43(0.32~0.57)	<0.001^*^	0.53(0.35~0.81)	0.003^*^
**Serum bilirubin(>1.2mg/l)**	1.36(1.07~1.72)	0.012^*^	1.14(0.86-1.51)	0.369	**Serum bilirubin(>1.2mg/l)**	1.61(1.25~2.07)	<0.001^*^	1.21(0.9~1.64)	0.2
**Serum cholesterol (>200mg/dl)**	0.95(0.7~1.31)	0.772	-	-	**Serum cholesterol (>200mg/dl)**	0.85(0.6~1.21)	0.366	-	-
**Serum triglyceride(>200mg/dl)**	0.85(0.49~1.48)	0.566	-	-	**Serum triglyceride(>200mg/dl)**	0.64(0.32~1.3)	0.216	-	-
**Serum AFP value(>400ng/ml)**	1.64(1.28~2.11)	<0.001^*^	1.5(1.1~2.05)	0.01^*^	**Serum AFP value(>400ng/ml)**	1.86(1.43~2.43)	<0.001^*^	1.78(1 2~2.46)	<0.001^*^
**Child-Pugh grade (B or C)**	2(1.5~2 66)	<0.001^*^	1.21 (0.82~1.79)	0.346	**Child-Pugh grade (B or C)**	2.63(1.98~3.51)	< 0.001^*^	1.71(1 13~2.59)	0.011^*^
**TNM stage**	1.43(1.26~1.61)	<0.001^*^	1.32(1.07~1.64)	0.011^*^	**TNM stage**	1.51(1.32~1.72)	<0.001^*^	1.29(1.02~1.62)	0.033^*^
**Diff. degree (Poor)**	1.39(1.16~1.68)	<0.001^*^	1.37(1.07~1.75)	0.012^*^	**Diff. degree (Poor)**	1.34(1.09~1.65	<0.005^*^	1.15(0 89~1.49)	0.284
**Tumor size (>5cm)**	1.9(1.52~2.37)	<0.001^*^	1.31(0.93~1.84)	0.124	**Tumor size (>5cm)**	2.1(1.65~2.66)	<0.001^*^	1.47(1.01~2.14)	0.045^*^
**Tumor number (multiplicity)**	1.32(1.01~1.73)	0.048^*^	0.99(0.71~1. 37)	0.933	**Tumor number (multiplicity)**	1.13(0.64~1.52)	0.434	-	-
**Vascular invasion (N/Y)**	0.74(0. 58~0.95)	0.02^*^	1.31(0.9~1.9)	0.165	**Vascular invasion (N/Y)**	0.7(0.53~0.92)	0.012^*^	1.18(0.55-0.97)	**0.398**

* P-value < 0.05

Interestingly, although the results showed that BMI < 25 seems to be a significant contributor for OS and RFS rates; according to Cox regression analysis, DM was not a potential risk factor for HCC prognosis after surgical treatment suggesting that there might be another factor(s) to influence BMI associated surgical outcome. Based on the analysis in Table 1, significantly lower percentage of DM and lower serum cholesterol level were detected in BMI^low^ group compared to BMI^high^ population, implying the potential correlation between these factors and BMI mediated contribution of post-operative survival rates. By using logistic regression analysis, BMI significantly indeed correlated with serum cholesterol levels (*P*-value = 0.029) indicating that pre-operative serum cholesterol level is an important factor in predicting OS and RFS rates in HCC patients undergoing curative hepatic resection surgery.

### Synergistic effect of BMI and serum total cholesterol levels for post-operative outcomes in HCC patients

Of 484 patients who received the operations, pre-operative serum cholesterol levels from 394 patients were recorded. Among them, 191, 30, 135 and 38 patients were classified into BMI^high^/Chol^high^ BMI^low^/Chol^high^, BMI^high^/Chol^low^ and BMI^low^/Chol^low^ groups, respectively. Further analysis showed no significant differences in age, gender, the preoperative liver function and histopathological parameters including the serum total bilirubin, serum albumin, serum AFP, Child-Pugh grade, TNM stage, differentiation status, tumor size, presence of vascular invasion and number of operated tumors among 4 groups (Table 3).

**Table 3:** Demographic data of HCC patients clarified with different BMI and Cholesterol level

Characteristics	BMI^low^/Cho^low^	BMI^low^/Cho^high^	BMI^high^/Cho^low^	BMI^high^/Cho^high^	P-value
**Age (years)**	62.4	59.9	62.4	58.7	0.211
**< 57**	57	11	38	18	
**≥ 57**	134	19	97	20	0.124
**Gender**					
**Male**	144	21	98	33	
**Female**	47	9	37	5	0.298
**DM status**					
**Non-DM**	147	22	89	27	
**DM**	44	8	46	11	0.182
**Platelet (x10^3^)**	157.3	164.9	141.4	169.8	0.106
**< 60000**	18	1	5	1	
**≥ 60000**	173	29	130	37	0.113
**Serum AFP (μg/L)**	3609.1	2840.3	1456.2	17471.6	0.12
**≤ 400**	146	23	107	30	
**> 400**	37	6	26	8	0.997
**Total bilirubin (mg/dl)**	1.02	0.94	1.39	0.93	0.478
**< 1.2**	140	24	96	28	
**≥ 1.2**	51	6	137	10	0.856
**Albumin (mg/dl)**	3.88	3.9	3.89	4.12	0.024^*^
**<3.5**	30	5	24	3	
**≥ 3.5**	158	25	109	35	0.515
**Sugar (mg/dl)**	106	112	122	109	0.485
**<126**	151	24	108	31	
**≥ 126**	31	6	25	7	0.93
**AST (IU/L)**	60	58.4	53.5	44.5	0.291
**< 40**	94	12	66	23	
**≥ 40**	96	8	69	15	0.4
**TG**	82	128	100	142	<0.001^*^
**< 200**	188	25	124	32	
**≥ 200**	2	5	6	5	<0.001^*^
**Child stage**					
**A**	148	20	109	34	
**B**	24	5	15	2	
**C**	2	0	0	0	0.512
**TNM stage**					
**I**	45	5	39	10	
**II**	75	14	55	20	
**III**	31	4	25	6	
**IV**	20	3	6	1	0.455
**Differentiation**					
**Well**	14	1	7	2	
**Moderate**	97	14	54	19	
**Poor**	58	10	61	16	0.248
**Size (cm)**					
**< 5**	128	19	102	24	
**≥ 5**	63	11	33	14	0.248
**Number**					
**Single**	157	27	115	31	
**Multiple**	34	3	20	7	0.674
**Vascular invasion**					
**Positive**	118	16	78	23	
**Negative**	51	7	42	11	0.854

* P-value < 0.05.

**Figure 2:**
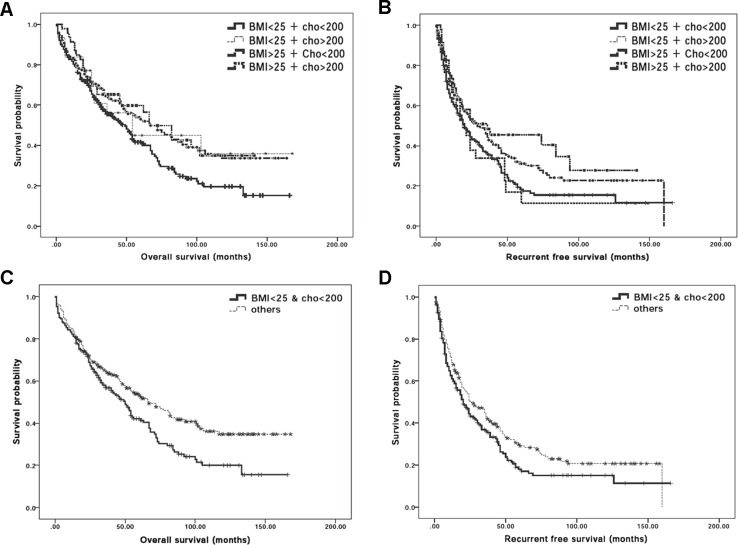
Synergistic effect of BMI and serum total cholesterol levels for post-operative outcomes in HCC patients **A.** Post-operative Kaplan-Meier survival curves for overall survival rate of HCC patients classified based on BMI = 25kg/m^2^ and serum cholesterol level = 200mg/dl. The cumulative overall survival rates showed significant low overall survival rate in BMI^low^/Chol^low^HCC patients who received hepatectomy when compared with BMI^high^/Chol^low^ HCC patients (*P*-value = 0.010). **B.** Post-operative Kaplan-Meier survival curves for disease survival rate of HCC patients classified based on BMI = 25kg/m^2^ and serum cholesterol level = 200mg/dl. The cumulative overall survival rates showed significant low disease free survival rate in BMI^low^/Chol^low^ HCC patients who received hepatectomy when compared with BMI^high^/Chol^low^ HCC patients (*P*-value = 0.031). **C.** Post-operative Kaplan-Meier survival curves for overall survival rate between the BMI^low/^Chol^low^ HCC patients and remaining HCC patients. The cumulative overall survival rates were significantly difference between these 2 groups (log-rank test; *P*-value = 0.007); **D.** Post-operative Kaplan-Meier survival curves for recurrence-free survival in BMI^low^/Chol^low^ HCC patients who received hepatectomy. The cumulative recurrence-free survival rates were significantly different between the BMI^low^/Chol^low^ and remaining HCC patients (log-rank test; *P*-value = 0.039).

To further determine whether differential cholesterol and BMI values exhibited different susceptibility for post-operative mortality and recurrence- related death, the OS and RFS rates after surgical treatment between different groups were analyzed. Among all 394 subjects, 232 (68.9%) patients died during the follow-up, comprising of 124 (64.9%) BMI^low^/Chol^low^, 15 (50%) BMI^low^/Chol^high^, 72(53.3%) BMI^high^/Chol^low^ and 17 (55.3%) BMI^high^/Chol^high^ patients. The OS rates at the 1^st^, 2^nd^, 3^rd^, 4^th^ and 5^th^ year were 82.2%, 68.8%, 56.8%, 50.6% and 41.5% for BMI^low^/Chol^low^ patients; 81.2%, 65%, 55.7%, 50.5% and 42.2% for BMI^low^/Chol^high^ patients; 83.7%, 72.3%, 66%, 59.6% and 54% for BMI^high^/Chol^low^ patients and 88.4%, 68.4%, 61.2%, 53.2% and 50.1% for BMI^high^/Chol^high^ patients. Kaplan-Meier curves for OS in BMI^low^/Chol^low^and other 3 groups indicated that the cumulative OS rates were significantly higher for BMI^high^/Chol^low^ patients compared with BMI^low^/Chol^low^ group (Figure 2A; log-rank test, *P*-value = 0.011). HCC recurred in 283 patients as 43 (74.8%) of them were BMI^low^/Chol^low^, 23 (79.3%) of them were BMI^low^/Chol^high^, 94 (69.6%) of them were BMI^high^/Chol^low^ and 23 (72%) of them were BMI^high^/Chol^high^. The RFS rates at the 1^st^, 2^nd^, 3^rd^, 4^th^ and 5^th^ year were 61%, 44.4%, 36.2%, 25.4% and 18% for BMI^low^/Chol^low^ group, 58.6%, 36.9%, 29.5%, 22.2% and 11.1% for BMI^low^/Chol^high^ group, 71.1%, 53.4%, 45%, 35.4% and 30% for BMI^high^/Chol^low^ group and 65.2%, 51%, 44.8%, 34.8% and 32.2% for BMI^high^/Chol^high^ patients. Kaplan-Meier curves for RFS rates showed significant difference in BMI^low^/ Chol^low^and the other 3 groups for the cumulative RFS rates (Figure 2B; log-rank test, *P*-value = 0.032). In brief, it could be concluded that in addition to liver functions and HCC tumor markers, lower BMI and lower preoperative serum cholesterol levels could also be important to determine postoperative outcomes in response to surgical procedure.

Based on previous analyses, BMI and serum total cholesterol levels could, solely or combined, predict post-operative outcomes. By using logistic Pearson's correlation analysis, BMI and serum cholesterol levels are indeed significantly correlated (*P*-value = 0.029). It therefore becomes important to further define whether BMI and serum cholesterol level is linked to predicate surgical outcomes in HCC patients. To this end, we determined the post-operative surgical outcomes, namely OS and RFS rates, between BMI^low^/Chol^low^ (*N* = 191) and remaining subjects (*N* = 203). For BMI^low^/Chol^low^ group, 232(58.9%) subjects died during follow-up; the respect OS rates at the 1^st^, 2^nd^, 3^rd^, 4^th^ and 5^th^ year were 82.2%, 68.8%, 56.8%, 50.6% and 41.5% as the OS rates for the remaining subject groups at the 1^st^, 2^nd^, 3^rd^, 4^th^ and 5^th^ year were 84.7%, 72.1%, 64.6%, 58.5% and 53.2%, respectively. Kaplan-Meier cumulative OS curves revealed a significant difference between these two groups (Figure 2C; log-rank test, *P*-value = 0.007). The RFS rates also significantly differed between BMI^low^/Chol^low^ and the rest of subjects. HCC recurred in 143 patients of the BMI^low^/Chol^low^ group and the RFS rates at the 1^st^, 2^nd^, 3^rd^, 4^th^ and 5^th^ year were 61%, 44.4%, 36.2%, 25.4% and 18%, respectively while the RFS rates in the non BMI^low^/Chol^low^ patients at the 1^st^, 2^nd^, 3^rd^, 4^th^ and 5^th^ year were 68.8%, 50.5%, 43.2%, 34.8% and 29.1%, respectively. Kaplan-Meier survival curves for RFS rates showed a significant difference between BMI^low^/Chol^low^ and others (Figure 2D; log-rank test, *P*-value = 0.039) implying that combination of low BMI and low serum cholesterol level might have advert effects on RFS.

Potential risk factors to influence OS rate and post-operative cancer recurrence rate of BMI^low^/Chol^low^ group were also examined. Using univariate analysis, 10 common factors significantly associated with OS and RFS rates were identified. They included: (1) the BMI^low^/Chol^low^ condition; (2) abnormal serum AST level; (3) hypoalbuminemia (serum albumin level < 3.5mg/dL); (4) higher serum AFP (AFP > 400mg/dl); (5) higher Child-Pugh grading; (6) higher TNM stage grading; and (7) greater tumor size ( > 5 cm) (Table 4). By using multivariate analysis, only (1) the BMI^low^/Chol^low^ condition and (2) hypoalbuminemia were significantly contribute to HCC recurrence (Table 4A). On the contrary, (1) the BMI^low^/Chol^low^ condition; (2) higher serum AFP (AFP > 400mg/dl); (3) higher Child-Pugh grading (with a Child-Pugh grade of B or C) and (4) greater tumor size ( > 5 cm) were defined as important regulator for OS rate (Table 4B).

**Table 4:** Univariate and multivariate Cox regression analysis for (A) OS and (B) RFS rates of BMI^low/^Chol^low^ HCC patients

A					B				
Variable	Univariate Cox-regression		Multivariate Cox-regression		Variable	Univariate Cox-regression		MultivariateCox-regression	
	HR(95% CI)	*P*-value	HR(95% CI)	*P*-value		HR(95% CI)	*P*-value	HR(95% CI)	*P*-value
**Age (>57yrs)**	1.3 (0.9~1.89)	0.16	-	-	**Age (>57yrs)**	1.23 (0.84~1.81)	0.291	-	-
**Gender (female)**	1.11(0.75~1.62)	0.61	-	-	**Gender (female)**	1.05(0.7~1.57)	0.829	-	-
**Diabetes(Y/M)**	1.04(0.71~1.53)	0.82	-	-	**Diabetes(N/Y)**	0.75(0.49~1.16)	0.198	-	-
**Serum AST value(>40IU/L)**	1.44(1.0~2)	0.032*	1.35(0.95~1.94)	0.097	**Serum AST value(>40IU/L)**	1.36(0.96~1.94)	0.087	-	-
**Sugar(>126 mg/dl)**	1.11(0.71~1.72)	0.66	-	-	**Sugar(>126 mg/dl)**	0.82(0.5~1.34)	0.429	-	-
**Serum albumin(>3.5 mg/dl)**	0.5(0.33~0.78)	<0.002^*^	1.53(0.3~0.92)	0.025*	**Serum albumin(>3.5 mg/dl)**	0.46(0.29~0.72)	0.001*	0.6(0.33~1.09)	0.094
**Serum bilirubin(>1.2 mg/dl)**	1.1(0.75~1.8)	0.63	-	-	**Serum bilirubin (>1.2 mg/dl)**	1.34(0.91~1.98)	0.142	-	-
**Serum AFP value (>400ng/ml)**	1.44(0.95~2.2)	0.088	-	-	**Serum AFP value (>400ng/ml)**	1.85(1.21~2.83)	0.005*	1.85(1.18~2.91)	0.007*
**Child-Pugh grade (B or C/A)**	1.78(1.17~2.71)	0.007^*^	1.27(0.75~2.14)	0.378	**Child-Pugh grade (B or C/A)**	2.6(1.72~3.93	<0.001^*^	2.24(1.32~3.78)	0.003*
**TNM stage**	1.29(1.07~1.54)	0.006*	1.2(0.94~1.52)	0.141	**TNM stage**	1.28(1.05~1.55)	0.013^*^	1(0.78~1.29)	0.976
**Diff. degree (Poor)**	1.2(0.91~1.59)	0.207	-	-	**Diff. degree (Poor)**	1.21(0.9~1.64)	0.209	-	-
**Tumor size (>5cm)**	1.56(1.1~2.2)	0.013*	1.42(0.87~2.33)	0.161	**Tumor size (>5cm)**	1.69(1.17~2.43)	0.005*	1.73(1.05~2.84)	0.032*
**Tumor number (multiplicity)**	1.06(0.69~1.61)	0.803	-	-	**Tumor number (multiplicity)**	0.96(0.61~1.51)	0.875	-	-
**Vascular invasion (N/Y)**	0.88(0.61~1.28)	0.51	-	-	**Vascular invasion (N/Y)**	1.04(0.7~1.53)	0.861	-	-

* P-value < 0.05.

## DISCUSSION

In the present study, we conducted a retro-perspective analysis for a cohort aiming to better define the role of BMI and its associated factors as HCC prognostic markers. The results showed that HCC patients with low pre-operative BMI, classified by BMI lower than 25kg/m^2^, exhibited lower OS and RFS rates after curative hepatic resection surgery. Further survival analysis indicated that lower preoperative serum cholesterol level in BMI^low^ patients could be an important contributor for poorer clinical prognosis. It is the first time, to the best of our knowledge, that a low BMI and low serum cholesterol levels were reported to contribute to poorer HCC prognosis after curative operation.

BMI has been considered an important factor in predicting the prognostic outcome in patients with colon, breast and lung cancers [22, 23]. In a physiological aspect, it was shown that visceral fat infiltration is associated with inferior oncologic outcomes following pancreatoduodenectomy for pancreatic adenocarcinoma providing a potential obesity associated mechanism for cancer promotion [24, 25]. In addition, high BMI has also been implicated as a risk factor for the development of postoperative complications after a variety of operations suggesting that BMI could be a useful marker to predict surgical outcome [26]. In HCC, some papers have shown that mortality and occurrence are associated with a high BMI [8, 27, 28] and others proposed that obesity is associated with increased morbidity and mortality following major abdominal procedures [7, 29]. Furthermore, a number of papers demonstrated an association between BMI and perioperative outcomes with hepatectomy [30-36] while other studies, however, found that overweight (BMI ≥ 25) is not a risk factor for postoperative surgical outcomes after hepatic resection in patients with primary HCC [13]. Taken together, it remains unclear whether obesity can be a prognostic factor after curative resection for primary HCC.

It was widely accepted that overweight and obese subjects could be more susceptible for higher cancer prevalence and poorer prognosis [8, 22, 23] whilst low BMI has never been considered as a prognostic factor contributing to post-operative OS or RFS rates in patients with neoplasias. In contrast, several results found that there was higher mortality in low BMI HCC patients compared with those with high BMI, possibly due to the consequences of poorer liver metabolism status in BMI^low^ subjects [13, 31]. To further clarify the controversial results between different studies, physiological data ranging from serological index, liver pathology (such as cirrhosis) to tumor states were analyzed to reveal potential underlying mechanisms. The analyses indicated that the BMI^high^ group had a higher total serum cholesterol value than BMI^low^ patients - in agreement with the fact that patients with metabolic disorders are more common in obese patients than in lean subjects [37]. Interestingly, combination of BMI and total serum cholesterol level served as a risk factor for surgical outcome but not for cancer recurrence prevalence by using Cox-regression analysis between BMI^low^/Chol^low^ and the rest of subjects. Nevertheless, the survival analysis showed a significantly poorer OS and RFS rates in the BMI^low^/Chol^low^ compared with the other subjects indicating that, in addition to liver function profile and AFP levels, tumor grade, serum cholesterol levels and BMI could be also important for determination of post-operative surgical outcome.

It is also noteworthy that the presence of DM was not a significant independent prognostic factor for RFS or OS rates in the current study using either univariate or multivariate analyses although a recent report concluded that obesity and DM promoted the risk of HCC [38]. Previous finding showed that insulin could induce peripheral lipolysis, hepatic accumulation of free fatty acid and production of reactive oxygen species (ROS) thereby leading to HCC development [39]. As most DM patients included in the current study were on their early disease stage and not on insulin medication, the null association between DM and RFS/OS is therefore expected.

The liver plays a central role in lipid metabolism involving the production and storage of apoproteins (Apo) and lipoproteins as well as catabolism of various lipids and excretion of cholesterol and phospholipids. An alteration in liver functions resulting from cellular injury often leads to changes in the serum concentration of cholesterol and lipoproteins. Low levels of plasma cholesterol and lipoproteins are often detected in chronic liver diseases and presented as a malnutritional and end stage liver function status. In molecular level, previous studies showed that down-regulation of acyl-transferase, a rate-limiting enzyme for lipoprotein synthesis, led to a decrease of serum triglyceride and total cholesterol levels in subjects with liver disease. In addition, an *in vitro* study further showed a deregulated synthesis of Apo A and/or reduced phosphatidylcholine (PC) as well as decreased levels of high-density, low-density, and very low-density lipoproteins (HDL, LDL, and VLDL, respectively). Furthermore, the blood lipids including triglycerides, total cholesterol, HDL, LDL, and VLDL, were negatively correlated with the severity of liver damage in patients with HBV- or HCV-mediated chronic liver dysfunction [40-42]. In addition, lower cholesterol level was correlated with severe liver fibrosis suggesting that low serum cholesterol levels could be often detected in diseased liver [40-43]. In the present study, it was found that lower total serum cholesterol level (*P*-value = 0.029) was detected in BMI^low^/Chol^low^ patients compared with the non BMI^low^/Chol^low^ subjects, indicating that higher recurrent rate and poorer OS rate in BMI^low^/Chol^low^ population could possibly be the consequences of imbalanced lipid metabolism, altered immune status and nutritional shortage of liver malfunction. Interestingly, no significant associations were found to correlate HBV/HCV infection (Supplementary Figure S1), liver fibrosis (Supplementary Figure S2) and DM (Supplementary Figure S3) with OS and RFS rates for HCC patients in current cohort implying that potential determinants for surgical outcomes in HCC patients remained to be determined.

In conclusion, this study showed for the first time that a low BMI and hypocholesteronemia contributed to lower OS or RFS rates. Coexistence of low BMI and low serum cholesterol level could serve as a promising prognostic factor to predict post-operative HCC recurrence and OS in patients undergoing surgical hepatectomy. The poorer prognosis in BMI^low^/Cho^low^ HCC patients undergoing hepatic resection surgery could be a consequence of poor peri-operative condition resulting from abnormal liver lipid metabolism. The finding could be of great benefit for surgeons to use pre-operative BMI and serum cholesterol levels as alternative index to achieve better surgical outcomes.

## MATERIALS AND METHODS

### Clinical information

Four hundred and eighty-four patients who had undergone curative hepatectomy for HCC between January 2000 and December 2010 at the Department of Gastroenterological Surgery, Ren-Ai branch, Taipei City Hospital, Taiwan were included in present study. The patients included in the present study were (i) with a good general condition with performance status test of grade 0 to 1, (ii) diagnosed with primary HCC, (iii) contained the tumor with a Child-Pugh classification grade of A or B, and (iv) exhibited the solitary or multiple HCCs those were clinically resectable and no abdominal computed tomographic (CT) or magnetic resonance imaging (MRI) evident vascular invasion or distant metastasis. In contrast, the patients (i) with microscopic cancer cell persistence at the surgical margin, (ii) having received multimodality therapy for multiple HCCs, (iii) detected with distant metastasis within 3 months after the operation, and (iv) lost to follow-up were excluded. Participants' body weights and heights were recorded before surgery in the in-hospital day as BMI was defined using the formula: weight (kg) divided by height (m) squared. According to the World Health Organization (WHO) classification [21], all participants were initially classified into 4 groups including the subjects with BMI < 18.5, 18.5 < = BMI < 25, 25 < = BMI < 30 and BMI > = 30 and showed no significant difference for OS and RFS rates among groups, except between groups of BMI < 18.5 and BMI > = 30 (Supplementary Figure S4). In addition, relative less cases of extreme BMI groups (*N* = 17 for BMI < 18.5 and *N* = 36 for BMI > = 30) and previous findings showing that overweight (25 < = BMI < 30) and obese (BMI > = 30) patients displayed similar postoperative outcome [44-46]; we therefore decided to divided all subjects into 2 groups of BMI < 25 and > 25 as BMI^low^ and BMI^high^, respectively. Further analysis according to the guideline from Laboratory Department, Taipei City hospital, Ren-Ai branch was also carried out. The patients were classified into four groups: BMI^high^/Chol^high^ BMI^low^/Chol^high^, BMI^high^/Chol^low^ and BMI^low^/Chol^low^ using 25 of BMI and 200mg/dl of serum cholesterol as cut-off values. The clinical data such as age, gender, blood glucose level, hepatitis infection, platelets count, serological alpha-fetoprotein (AFP) level, albumin, aspartate aminotransferase (AST), total bilirubin, total cholesterol, triglyceride and tumor tissue score, based on size, number, Child/TNM stage, differentiation status and vascular invasion were recorded. Postoperative mortality was defined as deaths within 30 days post-surgery as postoperative morbidity was defined as any complication requiring intervention during the perioperative hospitalization.

All patients included in this study were confirmed for HCC diagnosis based on pathological examination and evaluation for their serological hepatitis viral titer, liver function/cirrhosis and tumor characteristics by CT pre- and post-operatively. The hepatic reserve was defined using platelet count and Child-Pugh classification [47] as patients' immunological states were assessed by the absolute counts of peripheral blood leukocytes and lymphocytes [48-50]. Routine blood tests were performed on the day of admission and 7 days postoperatively. Tumorous parameters including tumor morphology and extent, serum alpha-fetoprotein levels, and portal vein thrombosis was classified based on the Union Internationale Contrele Cancer (UICC) classification [51, 52]. Patients were subjected to monthly follow-up physical examinations after operation while blood samples were collected to monitor serum AFP level at every re-visits. In addition, serial CT or liver ultrasonography was also performed every 3 to 6 months at re-visits to examine any possible recurrence.

### Statistical analysis

All statistical analyses were performed using SPSS 17.0 (SPSS, Inc. Chicago, IL, USA). The differential clinicopathological variables between the BMI^low^ and BMI^high^ groups were compared. The continuous variables are expressed as the mean ± standard deviation and comparisons were made using student's *t*-test as categorical variables were compared using chi-square tests. The OS and RFS rates were estimated by the Kaplan-Meier analysis and the differences in survival rate between groups were determined using the log rank test. The significant variables were analyzed in univariate and multivariate analysis using Cox's proportional hazard model while Cox regression was adopted for multivariate analysis of prognostic predictors. A *p-*value < 0.05 was considered to be significant.

## SUPPLEMENTARY FIGURES AND TABLES


