# Supplementary material for: Body mass index and cholesterol level predict surgical outcome in patients with hepatocellular carcinoma in Taiwan - a cohort study
Source: Oncotarget. 2016 Mar 24;7(16):22948–59. doi: 10.18632/oncotarget.8312 (PMC5008414; doi:10.18632/oncotarget.8312)
Supplement: Supplementary file 1 [file oncotarget-07-22948-s001.pdf]

Body mass index and cholesterol level predict surgical outcome in patients with hepatocellular carcinoma in taiwan - a cohort study

Supplementary Material

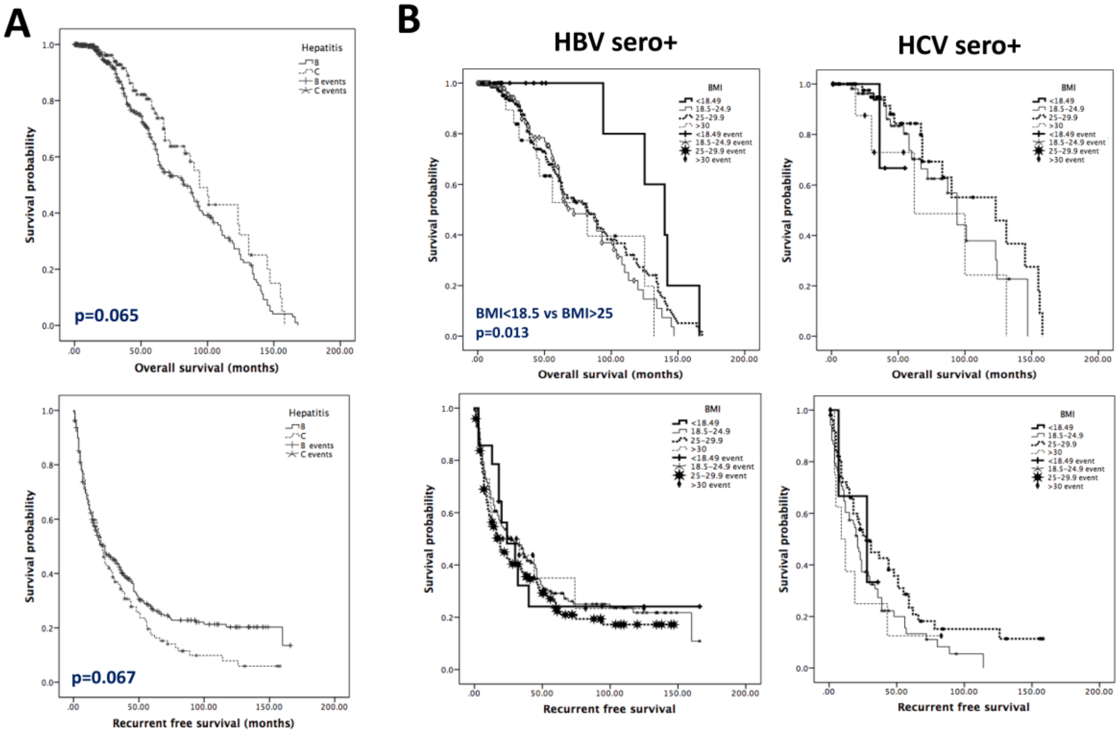

Supplementary Fig S1

(A) Kaplan-Meier survival curves for overall survival and recurrence-free survival rates in HCC patients with HBV and HCV infections.

(B) Cumulative overall survival and recurrence-free survival rates in HBV- and HVC-infected HCC patients classified according to BMI.

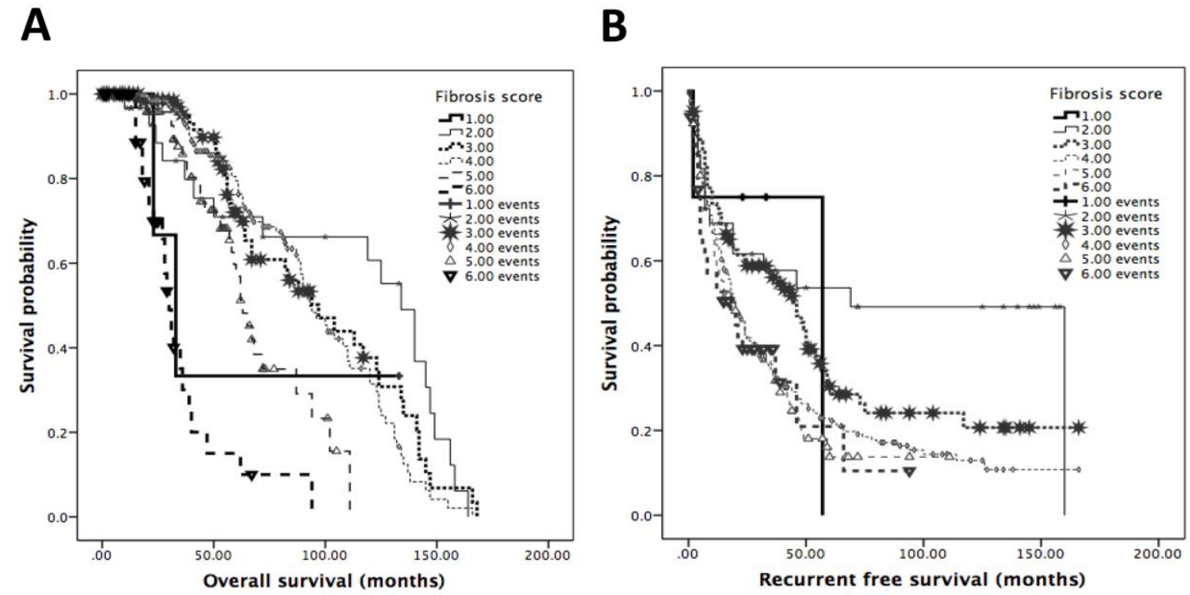

Supplementary Fig S2

Kaplan-Meier survival curves showed no significant correlation for (A) overall survival and (B) recurrence-free survival rates in HCC patients with different degree of liver fibrosis according to HAI classification.

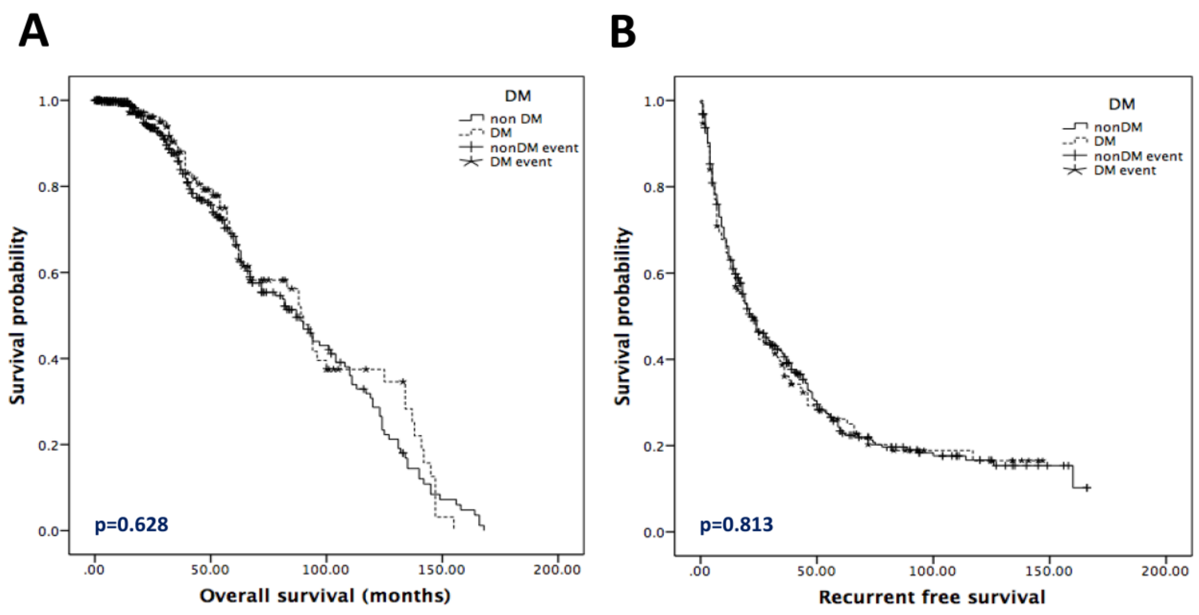

Supplementary Fig S3

Kaplan-Meier survival curves showed no significant correlation for (A) overall survival and (B) recurrence-free survival rates in HCC patients with and without DM.

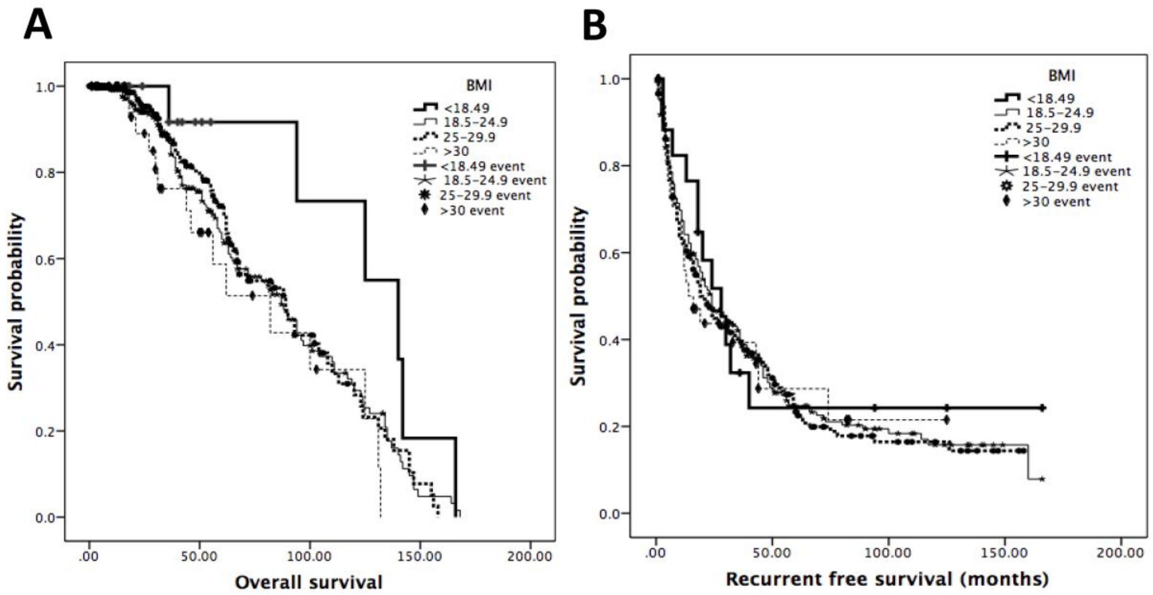

Supplementary Fig S4

Kaplan-Meier survival curves for (A) overall survival and (B) recurrence-free survival rates in HCC patients with different BMI according to WHO classification.
